# Photophysical and Computational Insights into Ag(I) Complexation of Porphyrinic Covalent Cages Equipped with Triazoles-Incorporating Linkers

**DOI:** 10.1021/acs.jpcb.2c01111

**Published:** 2022-04-28

**Authors:** Daniel Sánchez-Resa, Isabella Daidone, Ryan Djemili, Sonia Adrouche, Stéphanie Durot, Valérie Heitz, Laura Zanetti-Polzi, Barbara Ventura

**Affiliations:** † Isituto ISOF-CNR, Via P. Gobetti 101, 40129 Bologna, Italy; ‡ Department of Physical and Chemical Sciences, University of L’Aquila, Via Vetoio (Coppito 1), 67010 L’Aquila, Italy; § Laboratoire de Synthèse des Assemblages Moléculaires Multifonctionnels, Institut de Chimie de Strasbourg, CNRS/UMR 7177, Université de Strasbourg, 67000 Strasbourg, France; ∥ CNR Institute of Nanoscience, Via Campi 213/A, 41125 Modena, Italy

## Abstract

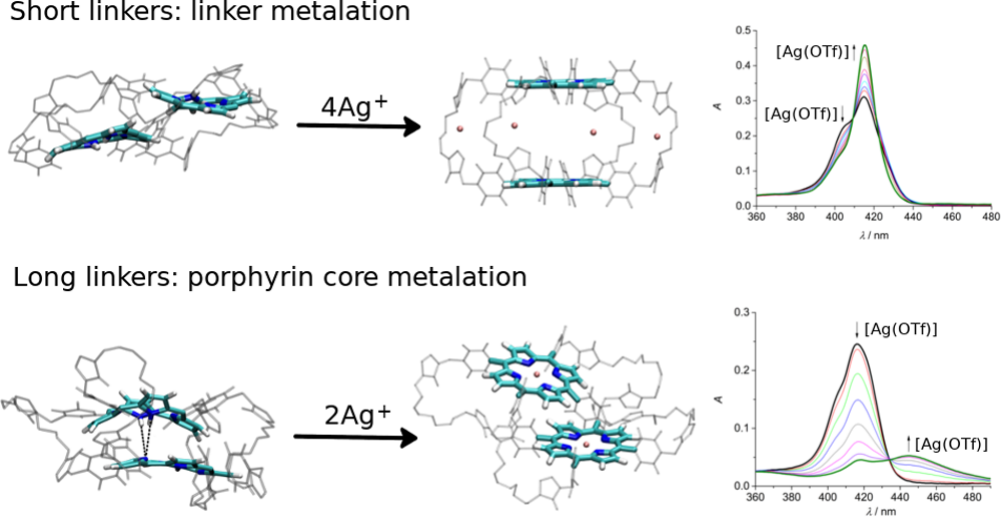

The photophysical characterization of four supramolecular complexes based on covalent cages **2H-S-2H**, **2H-L-2H**, **Zn-S-2H**, and **Zn-L-2H**, consisting in either two free-base porphyrins or one Zn(II) porphyrin and one free-base porphyrin connected by four flexible linkers of different lengths incorporating triazole binding sites, and their Ag(I) complexation are reported. The complexation processes have been followed by means of absorption and emission spectroscopies, and a comprehensive computational study explains the behavior of the free-base porphyrin-containing cages. Absorption and emission features have been interpreted on the bases of conformational changes, metalation processes, and modification of energy transfer efficiencies occurring in the different cases. In all cages, except **2H-L-2H**, the coordination of four Ag(I) ions to the lateral triazole groups of the linkers leads to the enlargement of their cavity. Only for **2H-L-2H** is a different behavior observed, where the process of silver metalation of the porphyrins’ core prevails.

## Introduction

The synthesis of molecular capsules as functional systems has attracted great interest in nanochemistry since these systems can work as molecular recognition systems, nanoreactors, or drug carriers, providing a confined environment that enhances guest stability, molecular reactivity, and catalysis.^1−3^

Among the different possibilities in the design of molecular cages, metalated or free-base porphyrins provide attractive architectures due to their chemical stability and their mimicry of natural chromophores.^1,4−19^

Porphyrin derivatives participate in natural processes for light harvesting, electron and energy transfer reactions, and catalysis or as oxygen transporters. Their stable aromatic core can be functionalized on meso or β pyrrolic positions, and the inserted metal can modulate their chemical, electronic, and photophysical properties. These systems are useful in various fields^20−22^ such as artificial photosynthesis, molecular electronics, molecular machines, catalysis, therapy, or surface engineering. The porphyrin unit can be considered as follows: (i) a large structural element that delineates the molecular cavity, (ii) an active component since its large π-delocalized core can stabilize π-conjugated guest molecules inside the cavity, whereas its metalated form can coordinate various ligands within the cage, and (iii) a redox and photoactive component, participating directly to the reactivity occurring inside the structure.^1^

In previous studies, we have shown that bis-Zn(II) porphyrin cages equipped with four flexible linkers incorporating peripheral 1,2,3-triazole binding sites allow for a control of the distance and orientation between the two Zn(II) porphyrins. Combined experimental and computational data showed that in solution, the cages adopt a flattened conformation based on π–π interactions between the porphyrins, whereas a large conformational change occurs upon binding of four Ag(I) ions to the peripheral ligands, leading to open cages with two cofacial porphyrins separated by about 9 Å.^23−26^ Thanks to their tunable cavity size, these cages were effective as allosteric receptors toward different kinds of guest molecules.^25,27^

The employment of silver(I) is very attractive due to its versatile coordination sphere and coordination numbers ranging from 2 to 6. This second row transition metal is widely used in supramolecular chemistry because silver–ligand bonds are labile in solution, allowing for the thermodynamic product to be reached via self-correction, and it is also easily decoordinated by the use of a compatible anion such as chloride^28−30^ or of light as an external stimulus.^31^

Later, we reported on bis-porphyrin cages constituted by either two free-base porphyrins or one Zn(II) porphyrin and one free-base porphyrin, connected by four flexible connectors that incorporate 1,2,3-triazole ligands linked with either an ethylene glycol unit or a diethylene glycol unit. The latter units confer different lengths to the connectors, leading to different possible conformations of the systems. Strong exciton interactions between the porphyrins occur due to the proximity of these units in the collapsed structure of the cages. In the monometalated cages, the free-base porphyrins showed altered fluorescence quantum yield and lifetime due to the presence of the closely spaced Zn counterpart. On the other hand, in these cages, the Zn-porphyrin emission is quantitatively quenched via a fast energy transfer process that sensitizes the free-base emission.^29^

In view of further exploring the properties of the four cages **2H-S-2H**, **2H-L-2H**, **Zn-S-2H**, and **Zn-L-2H**, here, we report on their complexation processes with Ag(I). A detailed photophysical study has been performed by means of steady-state absorption and emission spectroscopies and time-resolved luminescence techniques, combined with a thorough computational analysis of the structural features of the free-base cages, namely, **2H-S-2H** and **2H-L-2H**. This study completes the reported characterization of Ag(I) complexation of the analogues bis-Zn(II) porphyrin cages.^26^

## Materials and Methods

### Absorption and Emission Spectroscopies and Photophysics

Spectroscopy-grade CH_2_Cl_2_ and MeOH were obtained from Merck and used as received. Silver trifluoromethanesulfonate [Ag(OTf)] was obtained from Sigma-Aldrich. The latter has been stored in argon in a sealed vial under dark and dry conditions. Ag(OTf) solutions were used fresh and kept in the dark during the measurements.

Absorption spectra were recorded using PerkinElmer Lambda 650 UV–vis and PerkinElmer Lambda 950 UV–vis–NIR spectrophotometers.

Emission spectra were collected using an Edinburgh FLS920 fluorimeter, equipped with a Peltier-cooled Hamamatsu R928 PMT (280–850 nm), and corrected for the wavelength-dependent phototube response.Titration experiments were performed via incremental addition of micro-aliquots of stock solutions of Ag(OTf) (10^–3^ to 10^–4^ M) to a solution of the molecular cage (5–8 × 10^–7^ M) or model **2H-alkyne** (1.2 × 10^–6^ M). The final added volume was kept below 10% of the total volume to avoid dilution. The experiments have been conducted avoiding light exposure of the solutions. All the titrations of the cages were characterized by an early step, where the addition of silver (5–10 equiv) caused no changes in both absorption and emission features, attributed to an initial disaggregation process. Addition of an excess of silver salt caused degradation of the compounds in all cases. Titration data have been analyzed using ReactLab Equilibria software^32^ to determine the association constants.

Fluorescence lifetimes in the nanosecond range were detected by using an IBH time correlated single photon counting apparatus with nano-LED excitation at 465 nm. Analysis of the decay profiles against time was performed using decay analysis software DAS6 provided by the manufacturer.

Fluorescence lifetimes in the picosecond regime were measured by means of a Hamamatsu SynchroScan streak-camera apparatus (C10910-05 main unit and M10911-01 SynchroScan unit) equipped with an ORCA-Flash 4.0 V2 charge-coupled device and an Acton spectrograph SP2358. As the excitation source, a Newport Spectra-Physics Solstice-F-1K-230 V laser system, combined with a TOPAS Prime (TPR-TOPAS-F) optical parametric amplifier (pulse width: 100 fs and repetition rate: 1 kHz)^9^ was used, tuned at 560 nm. To reduce photo-degradation, the pump energy on the sample was reduced to 26 μJ/pulse. Emission from the sample, collected at a right angle with a 1 mm slit, was focused by means of a system of lenses into the spectrograph slit. Streak images were taken in the analog integration mode (100 exposures, exposure time: 2 s). The decays were measured over emission spectral ranges of 20–40 nm. HPD-TA 9.3 software from Hamamatsu was used for data acquisition and analysis. The overall time resolution of the system after deconvolution procedure was 1 ps.

### Molecular Dynamics Simulations

Molecular dynamics (MD) simulations of **2H-S-2H** and **2H-L-2H** are performed in CH_2_Cl_2_/MeOH (9:1) to mimic the experimental conditions. The initial structures of **2H-S-2H** and **2H-L-2H** were obtained from previous MD simulations of two covalent cages, **Zn-S-Zn** and **Zn-L-Zn**, consisting of two zinc porphyrins connected by the same four flexible spacers in the same solvent.^26^ Each Zn(II) ion is replaced with two protons to obtain a starting structure for the two free-base porphyrin cages.

MD simulations are performed using the GROMACS software package using the GROMOS force field. The GROMOS force field parameters for **2H-S-2H** and **2H-L-2H** were obtained from the Automated Topology Builder database.^33^ These parameters already proved their reliability in the MD simulations of the similar structures **Zn-S-Zn** and **Zn-L-Zn** and their corresponding silver(I)-complexed cages.^26,27^ The **2H-S-2H** and **2H-L-2H** cages are placed in a dodecahedral box large enough to contain the molecule and at least 1.0 nm of solvent on all sides with the appropriate number of CH_2_Cl_2_ molecules to reproduce the density of CH_2_Cl_2_ at 300 K and 1 bar (1.33 g cm^–3^). A proper number of CH_2_Cl_2_ molecules was then substituted with the corresponding number of MeOH molecules to reproduce the experimental CH_2_Cl_2_/MeOH (9:1) proportion. The force field parameters for CH_2_Cl_2_ and MeOH are obtained from the GROMACS topology database.

Simulations are carried out in the *NVT* (*i.e.*, with constant number of molecules, volume, and temperature) ensemble at a constant temperature of 300 K using the velocity rescaling temperature coupling.^34^ The LINCS algorithm^35^ is used to constrain bond lengths, and a time step of 2 fs for numerical integration of the equations of motion is used. The particle mesh Ewald method^36^ is used for the calculation of the long-range interactions, and a cutoff of 1.1 nm is used. After solute optimization and the subsequent solvent relaxation, each system is gradually heated from 50 to 300 K using short MD simulations. The trajectories are then propagated for 100 ns for each system. Coordinates are saved at every 1 ps.

## Results and Discussion

### Characterization of the Ag(I) Complexation Processes in Solution

The experiments have been carried out in 5–8 × 10^–7^ M CH_2_Cl_2_/MeOH (9:1) solutions of the cages by adding, as a Ag(I) ion source, Ag(OTf) salt diluted in the same solvent mixture.

**Figure 1:**
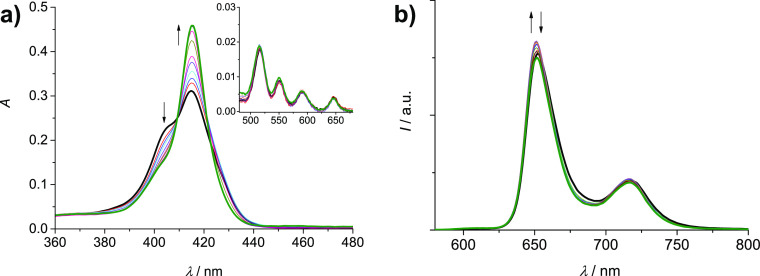
Absorption (a) and uncorrected emission spectra (λ_exc_ = 409 nm, isosbestic point) (b) of DCM/MeOH (9:1) solutions containing **2H-S-2H** (7.5 × 10^–7^ M) and increasing amounts of Ag(OTf) (0–45 equiv). Inset of (a): amplification of the Q-bands region (480–680 nm).

**Figure 2:**
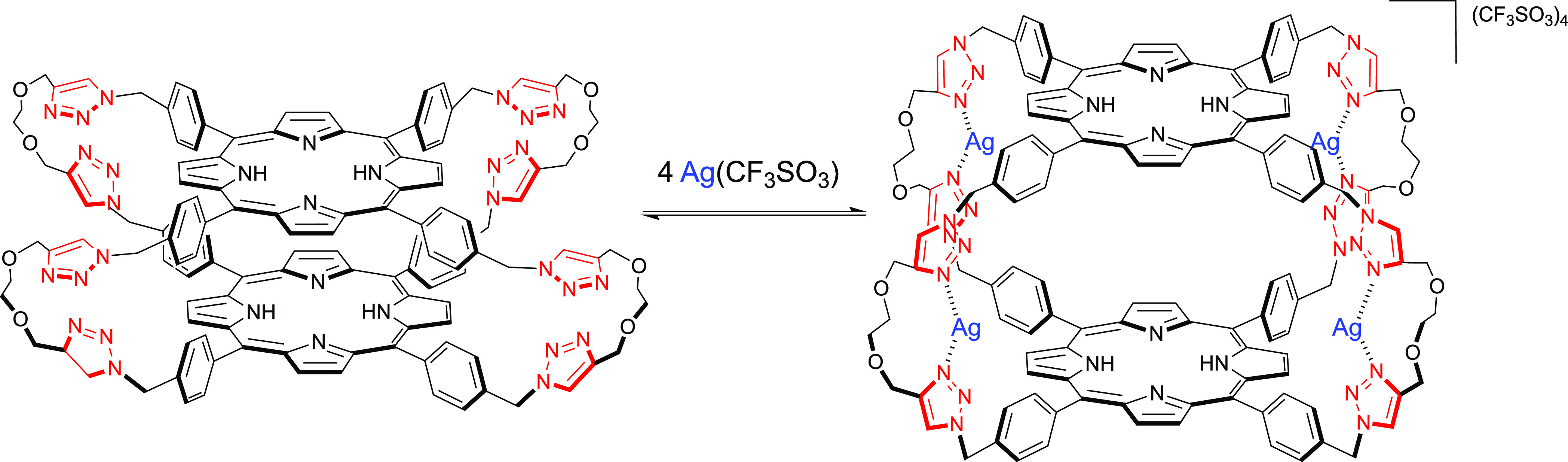
Schematic illustration of the complexation process of **2H-S-2H** with silver(I).

Addition of increasing amounts of Ag(I) to **2H-S-2H** (7.5 × 10^–7^ M) in the range 0–45 equiv causes absorption changes, as shown in Figure 1a. While the Q-bands region remains almost unaltered, the Soret band shows important variations. The peak at 415 nm increases in intensity, whereas the original splitting, due to exciton coupling between the porphyrin units, disappears (Figure 1a). The result is a Soret band similar to that reported for the free-base porphyrin model **2H-alkyne**.^29^ The absorption data support the hypothesis of a process of cage opening due to the coordination of the Ag(I) ions to the lateral triazoles since the larger distance between the units in the open conformation reduces the extent of the coupling, as also shown by previous calculations of the absorption spectra of analogous Zn-porphyrinic cages^26^ using a hybrid quantum/classical approach.^37−39^ The emission spectrum of the cage, collected upon excitation at 409 nm, the isosbestic point for the absorption titration, is slightly affected by the addition of Ag(I), and only a small increase in intensity followed by a minor decrease is observed (Figure 1b). The excited state lifetime measured at the end of the titration is only slightly reduced with respect to that of the pristine cage (8.4 *vs* 9.0 ns).^29^ The complexation process proposed for **2H-S-2H** is illustrated in Figure 2.

The absorption and emission data have been analyzed using software ReactLab Equilibria 1.1^32^ by considering a 1:4 (cage/Ag^+^) binding model and Ag^+^ as a non-absorbing and non-emissive species. The fitting converges, and the spectra generated by the software for the [Ag_4_(**2H-S-2H**)]^4+^ complex match well the experimental ones obtained at the end of titration (Figure S1). The derived average association constant, expressed in log(*K*_a_/M^–4^), is (18.94 ± 0.02).

**Figure 3:**
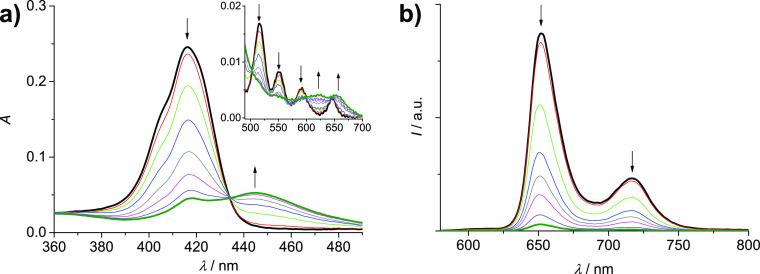
Absorption (a) and uncorrected emission (b) spectra (λ_exc_ = 434 nm, isosbestic point) of DCM/MeOH (9:1) solutions containing **2H-L-2H** (5.6 × 10^–7^ M) and increasing amounts of Ag(OTf) (0–30 equiv). Inset of (a): amplification of the Q-bands region (490–700 nm).

**Figure 4:**
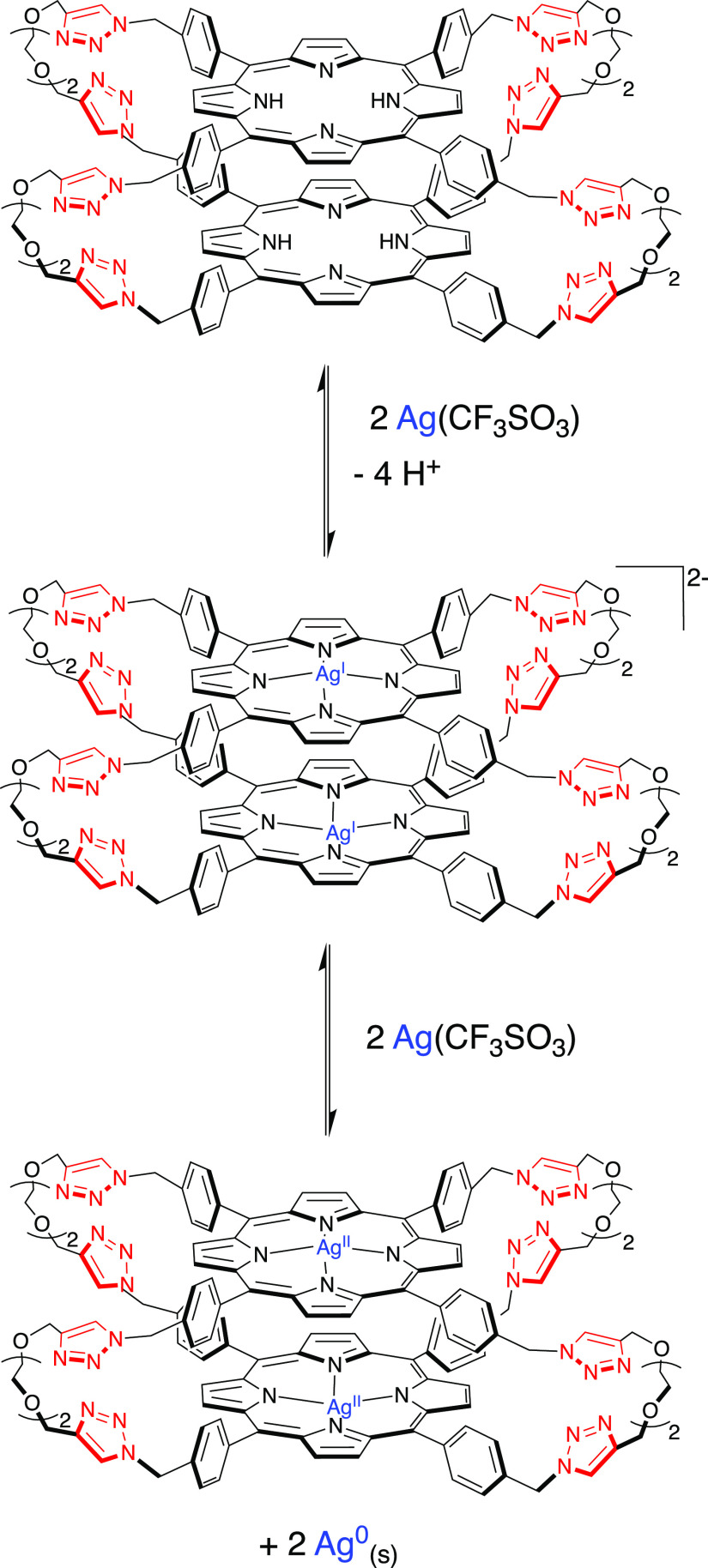
Schematic illustration of the metalation process of **2H-L-2H** with silver(I).

Differently, Ag(I) complexation of the analogous “long” cage **2H-L-2H** (5.6 × 10^–7^ M) in the range 0–30 equiv causes different and more drastic changes in absorption and emission spectra. Concerning the absorption features (Figure 3a), the Soret band at 416 nm almost disappears, while a new band at 445 nm increases (isosbestic point at 434 nm). In the Q-bands region, the original bands at 516, 550, 592, and 646 nm change into two new bands at 623 and 654 nm (isosbestic points at 503, 565, 580, and 599 nm). The observed changes point to a Ag(I) metalation process of the core of the free-base porphyrins, with the formation of a cage where both porphyrins are monometalated (Figure 4). The features of the species that forms, in particular the two Q bands at 623 and 654 nm, in fact, can be ascribed to a monometalated porphyrinic species, via comparison with the spectral features reported for [Ag(I)(TPP)]^−^ (bands at 457, 616, and 667 nm), obtained via electrochemical reduction from Ag(II)TPP.^40^ The formation of monometalated Ag(I) porphyrins is a quite unusual process, with respect to the more common bis-metalation that leads to Ag(I)_2_-porphyrin species,^43^ where the two metal ions are coordinated on the two sides of the porphyrin plane. The lack of formation of Ag(I)_2_-porphyrins in **2H-L-2H** can be attributed to the closed conformation of the cage that renders the inner face of the porphyrins barely accessible to the Ag(I) ions. Interestingly, the observed features are different also from those reported by us for the bis-metalation of a monomeric tetrapyridyl-functionalized free-base porphyrin.^41^ The presence of triazole groups in the linkers of **2H-L-2H** as weak bases close to the porphyrins, moreover, can favor the Ag(I) complexation step that involves deprotonation of the porphyrin core. Theoretical investigations (reported below) support the hypothesis of a Ag(I) metalation process of the core of the free-base porphyrins and discuss the reasons for the unusual behavior of **2H-L-2H** upon Ag(I) complexation. The emission titration, performed upon excitation at the isosbestic point, shows a progressive decrease of the fluorescence of the cage (Figure 3b), supporting the formation of metalated porphyrins, which are known to be weakly emissive with respect to their free-base counterparts.^42^ The luminescence lifetime at the end of the first step of titration was found to be 8.4 ns, which is thus only slightly decreased with respect to the value of 9.0 ns measured for the original cage^29^ and attributable to the residual fluorescence of the nonmetalated cages, likely interacting with silver(I) in their lateral groups.

It is worth noting that the further addition of Ag(I), up to 400 equiv, produces additional changes in the absorption spectrum, with the growth of two bands at 421 and 541 nm, while the fluorescence continues to decrease (Figure S2). A single Q band at *ca.* 540 nm is typical of Ag(II)-porphyrins,^43,44^ and this second process can be ascribed to the disproportionation of the Ag(I)-porphyrins of the cage to Ag(II)-porphyrins, with precipitation of Ag(0) (Figure 4), a process that often accompanies the complexation of silver with porphyrins since the latter, as ligands, can stabilize high-oxidation states of metal ions.^43,44^

The absorption and emission titration data comprehensive of both equilibria are quite complex to be treated using association fitting models. The data relative to the first process were analyzed using a 1:2 (cage/Ag^+^) model, with moderately good fitting results (Figure S3). The difficulty of this analysis is due to the fact that the Ag(I) metalation process is not completed, because of the disproportionation event that sums, and that lateral complexation of Ag(I) in the free-base cages, even if in a minor extent, cannot be excluded. An average association constant of log(*K*_a_/M^–2^) = (10.48 ± 0.01) is derived.

In order to obtain more insights in the complexation behavior of the two bis free-base porphyrin cages, model **2H-alkyne** (Chart S1) has been titrated with AgOTf in the same solvent mixture. The absorption titration shows two distinct families of curves (Figure S4a,c): up to 9 equiv, the decrease and broadening of the Soret band are observed, with the appearance of a shoulder at higher energy and a tail at lower energy, and in the Q-bands region a slight decrease of intensity of the bands occurs; in the range 9–30 equiv, the Soret band further reduces and slightly red-shifts, with the appearance of a new band centered at 465 nm, while in the Q-bands region, a further decrease of the original bands is observed with the formation of bands at *ca.* 625 and 653 nm. The changes observed in the first step point to the formation of assemblies constituted by two porphyrins held together by the coordination of Ag(I) ions to the appended triazole units.^28,45^ Conversely, the second process can be attributed to the metalation of the porphyrin cores in the formed assemblies, similarly to what observed for **2H-L-2H**. Moreover, the addition of an excess of silver leads to a degradation/disproportionation process (Figure S4e). As regards the emission, a progressive decrease of intensity is observed in all phases (Figure S4b,d,f), compatible with (i) a quenching interaction among the Ag(I) ions and the porphyrins upon the formation of the assemblies in the first step, (ii) formation of non-emissive Ag(I) porphyrinic species in the second step, and (iii) degradation/disproportionation processes in excess of silver.

The results evidence how the different behaviors of the two cages **2H-S-2H** and **2H-L-2H** toward Ag(I), where the lateral coordination and the core coordination prevail *versus* the other process, respectively, depend uniquely on the different lengths of the linkers and conformations of the cages, as discussed in detail in the Computational Results Section.

**Figure 5:**
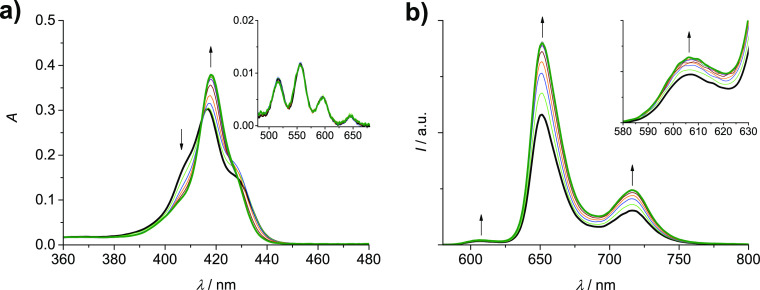
Absorption (a) and uncorrected emission spectra (λ_exc_ = 415.5 nm, isosbestic point) (b) of DCM/MeOH (9:1) solutions containing **Zn-S-2H** (5.5 × 10^–7^ M) and increasing amounts of AgOTf (0–40 equiv). Inset of (a): amplification of the Q-bands region (480–680 nm). Inset of (b): amplification of the 580–630 nm region.

**Figure 6:**
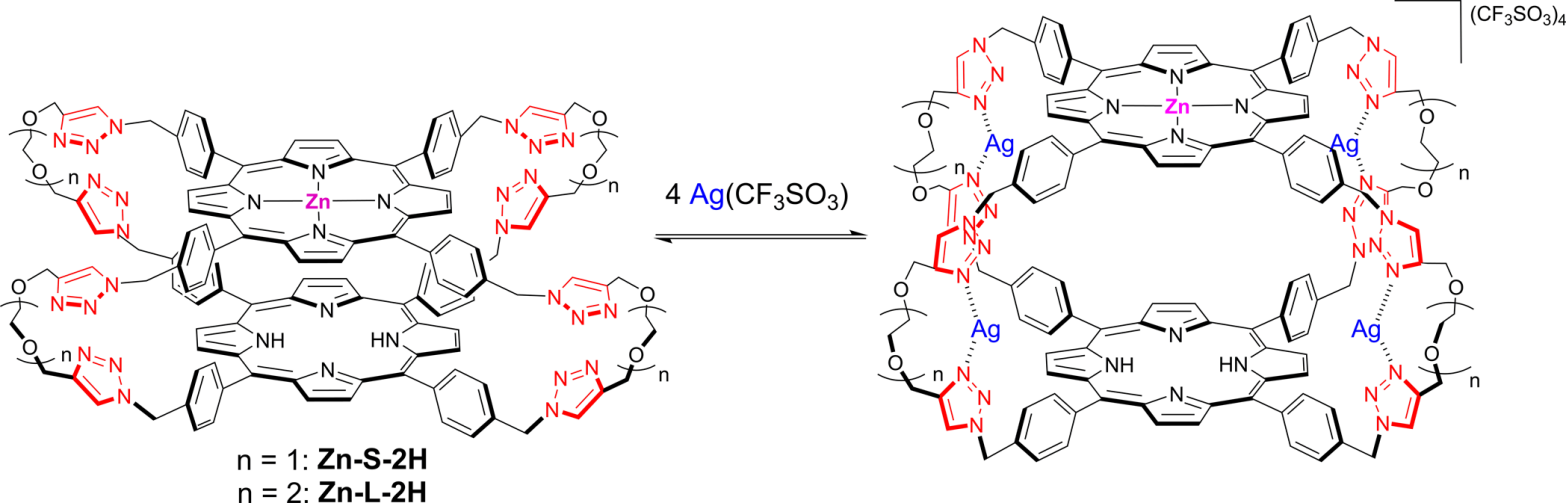
Schematic illustration of the complexation process of **Zn-S-2H** (*n* = 1) and **Zn-L-2H** (*n* = 2) with silver(I).

Addition of increasing amounts of Ag(I) to the monometalated cage **Zn-S-2H** (5.5 × 10^–7^ M) in the range 0–40 equiv causes changes both in absorption and emission features. The absorption spectrum evolves with the reduction of the splitting of the Soret band, resulting in the formation of a single band with maximum at 418 nm (Figure 5a), while the Q-bands region remains almost unaltered. The data point toward a process of cage opening, similarly to what is occurring in the parent **2H-S-2H** cage. The emission behavior is characterized by an increase in intensity of both the bands of the free-base unit (652 and 717 nm)^29^ and of the Zn-porphyrin (606 nm, the second band at *ca.* 660 nm is superimposed with the free-base porphyrin emission)^29^ (Figure 5b). The increase of the Zn-porphyrin emission is more evident by subtracting the free-base contribution from each spectrum of the cage, calculated by normalizing the emission spectrum of **2H-S-2H** at 712 nm (Figure S5). These data can be explained considering the effect of the cage opening process on the emission features of both units. We previously showed that the emission properties of the free-base component in **Zn-S-2H** are strongly affected by the presence of the closely spaced Zn-porphyrin counterpart, with the result of an almost halved quantum yield and a reduced lifetime (6.7 *vs* 8.5) with respect to the model monomer and cage **2H-S-2H**, attributed to a change in the molecular symmetry or to an increased intersystem crossing rate induced by the close proximity of the Zn center.^29^ The recovery of the free-base emission intensity observed upon the addition of Ag(I) testifies the separation of the two units, following the complexation of the Ag(I) ions with the lateral triazole groups (Figure 6). Indeed, the lifetime of the free-base emission measured at 720 nm for the complex was found to be 7.9 ns, which is closer to the value of 8.5 ns for the free-base porphyrin unaffected by the Zn counterpart.^29^ The parting of the two units toward an open cage conformation also decreases the efficiency of the energy transfer occurring from the Zn-porphyrin to the free-base counterpart,^29^ with the result of an increase of the emission intensity of the Zn component in the complexed cage.

The titration data could be satisfactorily fitted using a 1:4 (cage/Ag^+^) binding model (Figure S6) that provided a value of log(*K*_a_/M^–4^) = (22.09 ± 0.05) for the association constant.

**Figure 7:**
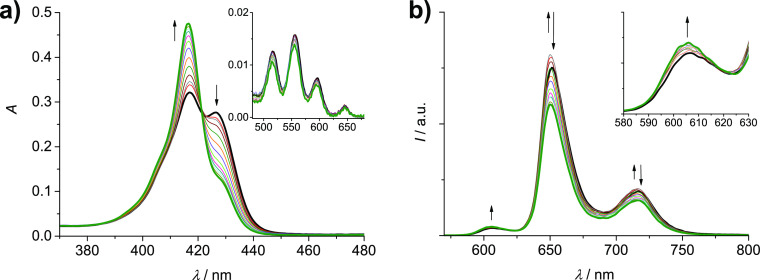
Absorption (a) and uncorrected emission spectra (λ_exc_ = 421 nm, isosbestic point) (b) of DCM/MeOH (9:1) solutions containing **Zn-L-2H** (6.4 × 10^–7^ M) and increasing amounts of AgOTf (0–25 equiv). Inset of (a): amplification of the Q-bands region (480–680 nm). Inset of (b): amplification of the 580–630 nm region.

Finally, addition of increasing amounts of Ag(I) to the “long” monometalated cage **Zn-L-2H** (6.5 × 10^–7^ M) in the range 0–25 equiv provokes changes in absorption and emission spectra, as shown in Figure 7. In absorption, a reduction of the splitting of the Soret band is observed with an increase of the band at 417 nm and a reduction of the shoulder at 426 nm (Figure 7a). In the Q-bands region, no significant modifications are observed. These spectral features again support a process of cage opening as in the previous cases (Figure 6). Concerning the emission spectra, a particular behavior is observed (Figure 7b): the band of Zn-porphyrin at 606 nm displays a constant increase in intensity, also considering the subtraction of the free-base contribution (Figure S7), while the bands of the free-base component at 652 and 716 nm show a slight increase, followed by a decrease. The gain in intensity of the Zn-porphyrin emission can be explained by the reduction of the efficiency of the energy transfer process occurring in the cage due to the increased distance between the two units, as in the parent **Zn-S-2H**. The dual behavior of the free-base emission is less straightforward: while the initial increase can be attributed to a recovery of the emission of the unit, which is reduced in the cage by the presence of the Zn counterpart (*ca.* 20% reduction),^29^ the following decrease seems to be due to an interaction of the free-base porphyrin with the linked Ag(I), while the metalation of the core can be excluded by the absorption features of the complex. The lifetime of the free-base component in the complex was found to be slightly decreased compared to that of the same unit in the bare cage, that is, 7.0 *versus* 7.6 ns.^29^

A value of log(*K*_a_/M^–4^) = (20.78 ± 0.02) was derived for the formation of the complexed cage (Figure S8).

**Figure 8:**
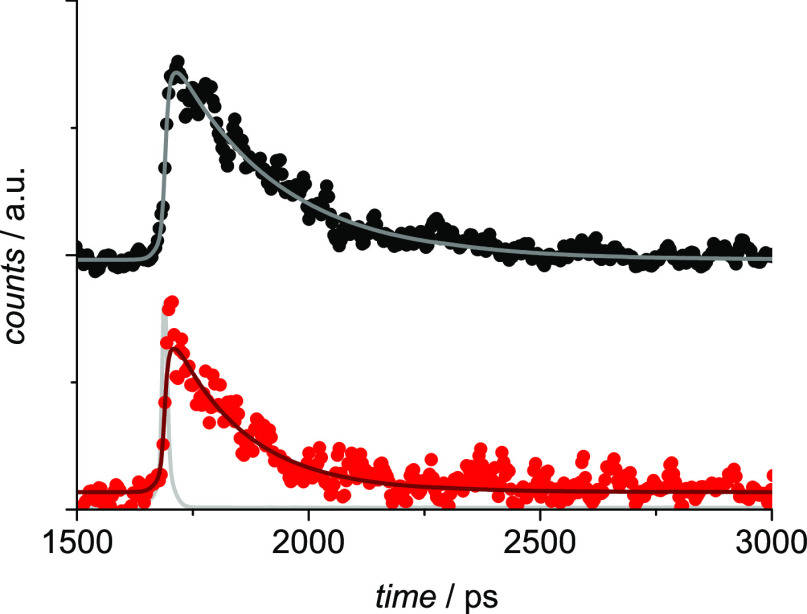
Luminescence decays in the 600–620 nm region for **Zn-S-2H** (black dots) and **Zn-L-2H** (red dots) added with 30 equiv of AgOTf. The biexponential fittings are reported as lines. The excitation profile is shown in light gray. Excitation at 560 nm (26 μJ/pulse).

In order to study in more detail the modification of the energy transfer process in the cages **Zn-S-2H** and **Zn-L-2H** upon the addition of Ag(I), ultrafast luminescence measurements on the cages added with Ag(I) were performed. Figure 8 shows the luminescence time profiles of the two complexed cages in the region 600–620 nm, where only emission from the Zn-porphyrin is collected. The decays are fitted with lifetimes of 230 and 160 ps for **Zn-S-2H** and **Zn-L-2H**, respectively. These lifetimes are longer than those measured in the pristine cages, that is, 10 and 7 ps, respectively.^29^ By considering a lifetime of 1.7 ns for the unquenched Zn-porphyrin model **Zn-alkyne**,^29^ the efficiency of the energy transfer is thus reduced from 99.4 to 86.5% in **Zn-S-2H** and from 99.6 to 90.6% in **Zn-L-2H**, accounting for the observed increase in the emission intensity upon titration with Ag(I) and in agreement with the absorption changes described above, showing increasing distances between the porphyrins upon titration with Ag(I). The observation that the distance between the porphyrins is larger in the complexes with Ag(I) than in the pristine cages was also previously demonstrated by means of a combined experimental and computational study on the analogous Zn-porphyrinic cages.^26^ Moreover, it is interesting to note that the lifetime of the Zn-porphyrin component in the “short” complexed cage is still longer than that of the same unit in the “long” and more flexible analogue, indicating a closer disposition of the two units in the latter, as established for the pristine cages and for the analogous Zn-porphyrinic cages studied previously.^26,29^

### Computational Results

In order to better understand the peculiar spectroscopic behavior of **2H-L-2H**, we performed MD simulations of both **2H-S-2H** and **2H-L-2H** in the absence of Ag(I) to compare the structural and dynamic features of the two cages in their uncomplexed state. The experimental results indeed suggest that the different length of the linkers can determine different closed conformations and, therefore, different reactivities of the two cages toward the silver ions.

**Figure 9:**
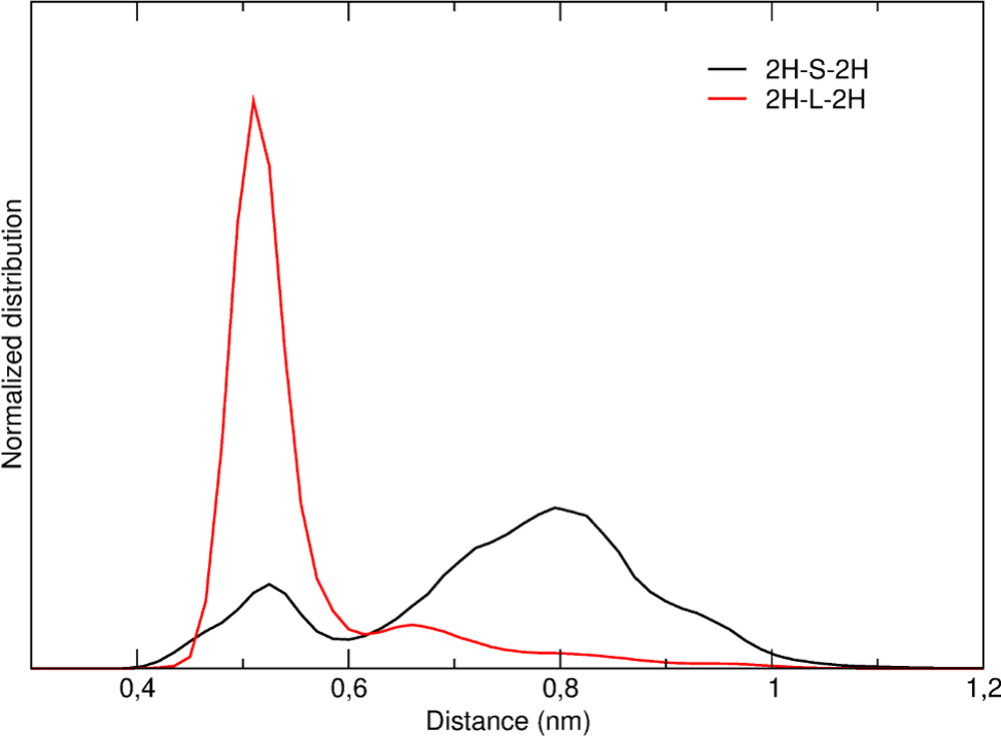
Normalized distribution of the distance between the centers of mass of the two porphyrin rings in the MD simulations of **2H-S-2H** (black) and **2H-L-2H** (red).

According to the MD simulations, the closed conformations sampled by the two cages are indeed rather different. In **2H-L-2H**, the distance between the porphyrin rings is on average lower than that in **2H-S-2H** (see Figure 9). In **2H-L-2H**, the distance between the centers of mass of the two porphyrin rings is essentially fixed at around 0.5 nm. In **2H-S-2H**, instead, the distribution of the distance between the two porphyrins is bimodal and features a minor low distance peak at 0.5 nm and a major broad high distance peak centered at ≈0.8 nm.

**Figure 10:**
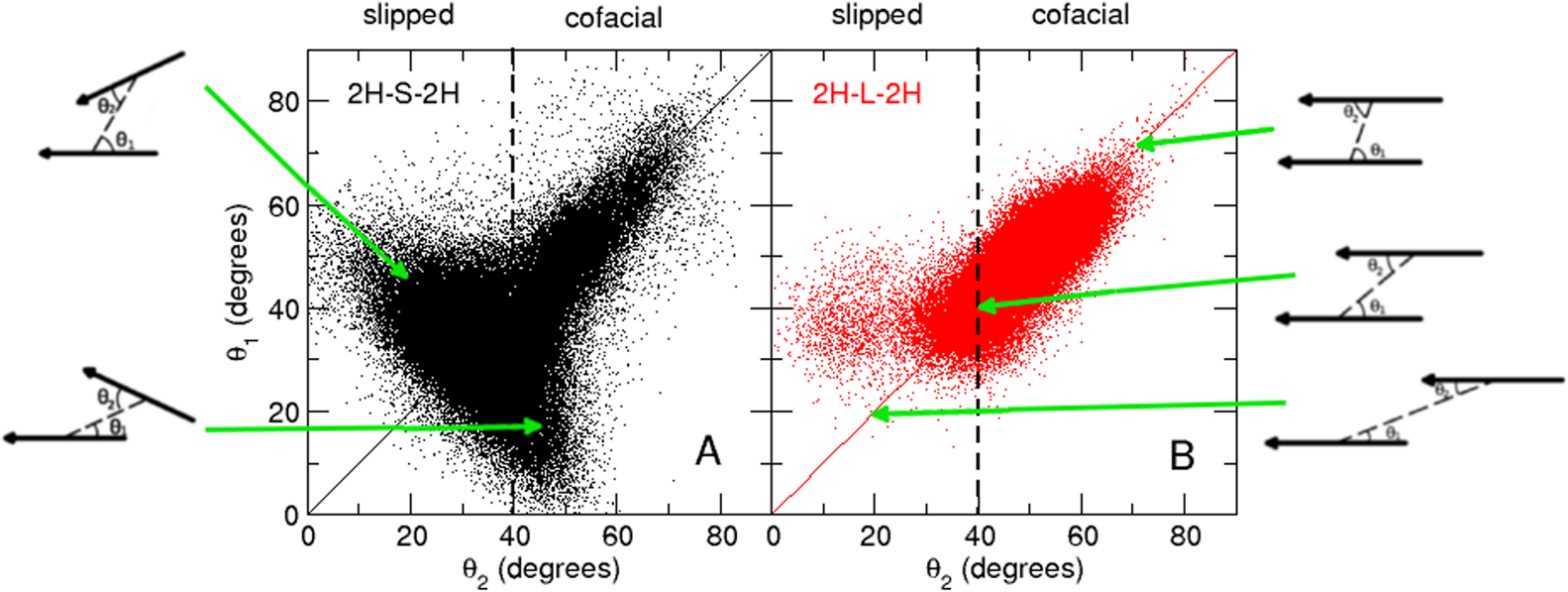
Bidimensional distribution of θ_1_ and θ_2_ in the MD simulations of **2H-S-2H** (black) and **2H-L-2H** (red). The relative orientation of the two porphyrin rings is also sketched.

Also, the relative orientations of the two porphyrin rings are different in the two cages. In Figure 10, we report the bidimensional distribution of θ_1_ and θ_2_, defined as the angles between each porphyrin plane and the vector connecting the two centers of mass. In **2H-L-2H**, the two porphyrin planes are essentially parallel (θ_1_ ≈ θ_2_) and cofacial (θ_1_, θ_2_ > 40°). In **2H-S-2H**, although parallel and cofacial configurations are also sampled, the two planes more often assume oblique (θ_1_ ≠ θ_2_) and slipped (θ_1_, θ_2_ < 40°) configurations. The parallel and cofacial configurations of **2H-S-2H** are explored when the two planes are at low distances (≈0.5 nm, see Figure S9). This suggests that for both **2H-S-2H** and **2H-L-2H**, at very low distances, parallel and cofacial relative orientations are favored. However, these configurations are much more frequent in **2H-L-2H**. This can be due to the fact that the shorter linkers, being more rigid, less easily accommodate very closed conformations that require a marked bending of the linkers.

**Figure 11:**
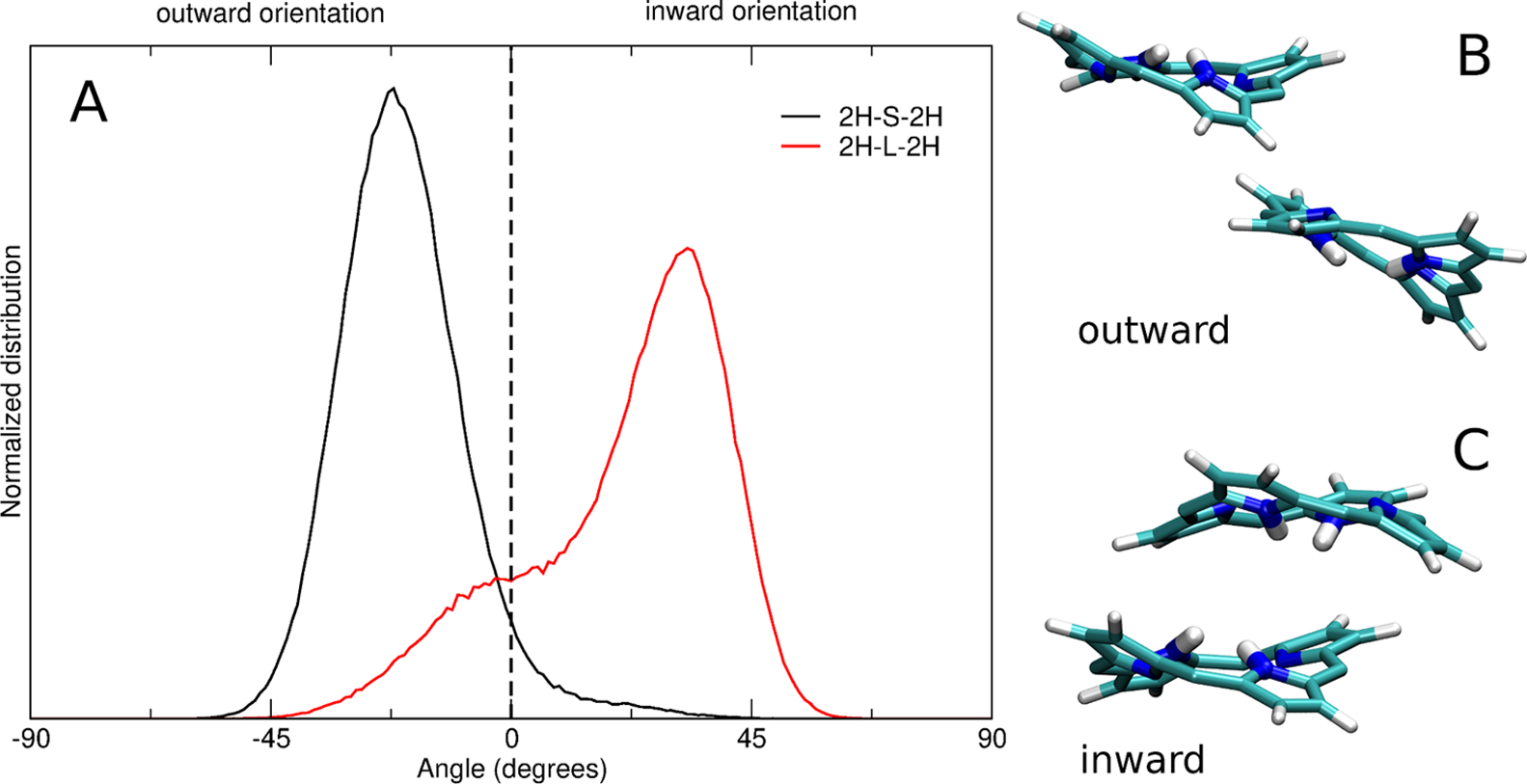
(A) Normalized distribution of the angle between the porphyrin NH groups and the porphyrin planes in the MD simulation of **2H-S-2H** (black) and **2H-L-2H** (red). The curve is obtained by averaging the distributions of the angles obtained for each of the four NH groups of the cage. Representative structures of the two porphyrin planes with the NH groups in the outward (B, **2H-S-2H**) and inward (C, **2H-L-2H**) orientations.

Interestingly, these different behaviors of the two uncomplexed cages can also affect their binding activity toward the silver ions. As shown in Figure 11, the more slipped conformations sampled by **2H-S-2H** determine an average “outer” orientation of the two NH groups of the porphyrin rings (*i.e.*, the two hydrogens point toward the solvent). On the contrary, in **2H-L-2H**, an “inward” orientation of the two NH groups is favored (*i.e.*, the two hydrogens point toward the cage cavity). This inward orientation determines the exposure to the solvent of the reactive lone pair of the nitrogen atoms. Upon the addition of Ag^+^ in solution, the nitrogen atoms can thus easily coordinate the silver ions, in competition with the complexation process involving the linkers.

**Figure 12:**
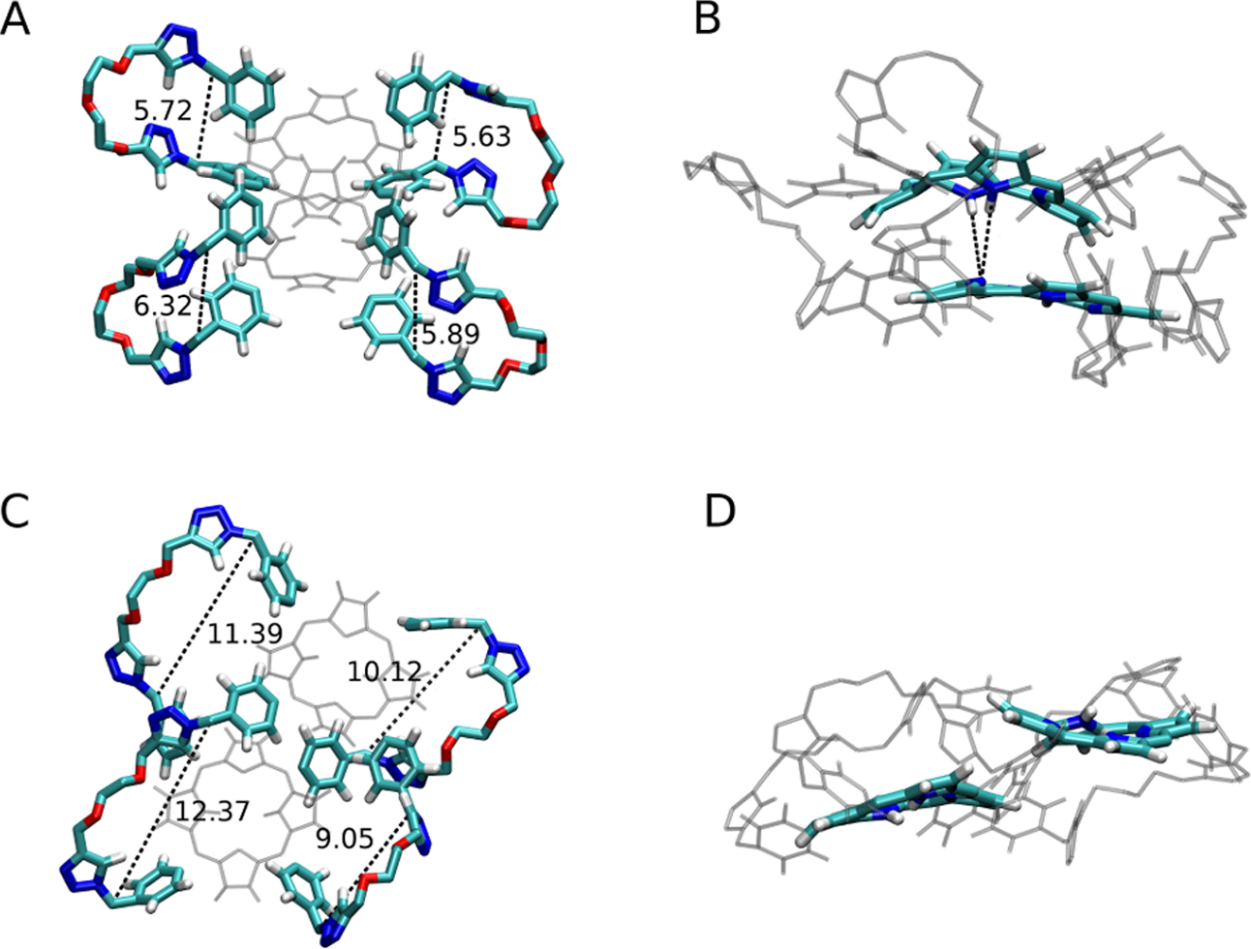
In **2H-L-2H**, the flexibility of the longer linkers allows their bending (A), with the porphyrin planes stacked in a parallel cofacial configuration, which in turn promotes a favorable interaction between the NH groups of one plane and the bare nitrogens of the other plane, as highlighted in (B). In **2H-S-2H**, the shorter linkers are less flexible and assume open configurations (C), forcing the two porphyrin planes in slipped configurations, in which the NH–N interactions are not possible (D). The distance between the two carbons connecting the phenyl and triazole rings in each linker is reported in angstroms in (A,C).

The stable inward orientation in **2H-L-2H** is driven by a favorable electrostatic interaction between the NH groups of one plane and the bare nitrogens of the other plane (see Figure 12B,D, in which representative structures of the inward and outward conformations are reported for **2H-L-2H** and **2H-S-2H**, respectively). These tightly packed structures require all the linkers to be bent: this is possible in **2H-L-2H** but much less probable in **2H-S-2H**, as shown in Figure S10 (see also Figure 12A,C). We also note that in **Zn-L-2H**, in which no evidence for the metalation of the free-base porphyrin is observed, the presence of the Zn center hinders the interaction between the NH groups of the free-base porphyrin and the nitrogens of the other porphyrin plane. This observation supports our explanation that these interactions, determining the “inward” orientation, play a major role in the metalation process of **2H-L-2H**.

## Conclusions

In this study, we have shown that the coordination of Ag(I) to the triazole ligands present in cages **2H-S-2H**, **Zn-S-2H**, and **Zn-L-2H** leads to the opening of their cavities within the range of 20–40 equiv. The changes in the absorption spectra point to a separation of the porphyrins since the reduction of the splitting of the Soret band confirms a reduction of the coupling between the units. The fluorescence changes are in agreement with the proposed mechanism. Instead, for cage **2H-L-2H**, a porphyrin metalation process, dominating upon the complexation of the lateral triazoles, is observed. By means of MD simulations, we ascribe this behavior to a more favorable orientation of the porphyrinic NH groups, if compared to its analogous **2H-S-2H**. In fact, differently from the cage with short linkers, in the cage with longer linkers, the NH groups point toward the interior of the cage, leading to the exposure of the reactive lone pair of the nitrogens to the solvent, possibly enhancing the reactivity toward the binding of the Ag(I) ions. This favorable orientation is allowed in **2H-L-2H** by the higher flexibility of the longer linkers.

## Supporting Information Available

Additional spectroscopic and computational data (PDF)

## References

[ref1] Durot S.; Taesch J.; Heitz V. Multiporphyrinic Cages: Architectures and Functions. Chem. Rev. 2014, 114, 8542–8578. 10.1021/cr400673y. 25026396

[ref2] Chakrabarty R.; Mukherjee P. S.; Stang P. J. Supramolecular Coordination: Self-Assembly of Finite Two- and Three-Dimensional Ensembles. Chem. Rev. 2011, 111, 6810–6918. 10.1021/cr200077m. 21863792 PMC3212633

[ref3] Mukhopadhyay R. D.; Kim Y.; Koo J.; Kim K. Porphyrin Boxes. Acc. Chem. Res. 2018, 51, 2730–2738. 10.1021/acs.accounts.8b00302. 30345738

[ref4] Balaban T. S. Tailoring Porphyrins and Chlorins for Self-Assembly in Biomimetic Artificial Antenna Systems. Acc. Chem. Res. 2005, 38, 612–623. 10.1021/ar040211z. 16104684

[ref5] Sprafke J. K.; Kondratuk D. V.; Wykes M.; Thompson A. L.; Hoffmann M.; Drevinskas R.; Chen W.-H.; Yong C. K.; Kärnbratt J.; Bullock J. E.; et al. Belt-Shaped Pi-Systems: Relating Geometry to Electronic Structure in a Six-Porphyrin Nanoring. J. Am. Chem. Soc. 2011, 133, 17262–17273. 10.1021/ja2045919. 21939246

[ref6] Griffith M. J.; Sunahara K.; Wagner P.; Wagner K.; Wallace G. G.; Officer D. L.; Furube A.; Katoh R.; Mori S.; Mozer A. J. Porphyrins for Dye-Sensitised Solar Cells: New Insights into Efficiency-Determining Electron Transfer Steps. Chem. Commun. 2012, 48, 4145–4162. 10.1039/c2cc30677h. 22441329

[ref7] Wytko J. A.; Ruppert R.; Jeandon C.; Weiss J. Metal-Mediated Linear Self-Assembly of Porphyrins. Chem. Commun. 2018, 54, 1550–1558. 10.1039/c7cc09650j. 29363684

[ref8] Hong S.; Rohman M. R.; Jia J.; Kim Y.; Moon D.; Kim Y.; Ko Y. H.; Lee E.; Kim K. Porphyrin Boxes: Rationally Designed Porous Organic Cages. Angew. Chem., Int. Ed. 2015, 54, 13241–13244. 10.1002/anie.201505531. 26305107

[ref9] Yu C.; Long H.; Jin Y.; Zhang W. Synthesis of Cyclic Porphyrin Trimers through Alkyne Metathesis Cyclooligomerization and Their Host-Guest Binding Study. Org. Lett. 2016, 18, 2946–2949. 10.1021/acs.orglett.6b01293. 27267936

[ref10] Hwang I.-W.; Kamada T.; Ahn T. K.; Ko D. M.; Nakamura T.; Tsuda A.; Osuka A.; Kim D. Porphyrin Boxes Constructed by Homochiral Self-Sorting Assembly: Optical Separation, Exciton Coupling, and Efficient Excitation Energy Migration. J. Am. Chem. Soc. 2004, 126, 16187–16198. 10.1021/ja046241e. 15584755

[ref11] Hernández-Eguía L. P.; Escudero-Adán E. C.; Pintre I. C.; Ventura B.; Flamigni L.; Ballester P. Supramolecular Inclusion Complexes of Two Cyclic Zinc Bisporphyrins with C 60 and C 70: Structural, Thermodynamic, and Photophysical Characterization. Chem.—Eur. J. 2011, 17, 14564–14577. 10.1002/chem.201101511. 22095593

[ref12] Li W.-S.; Kim K. S.; Jiang D.-L.; Tanaka H.; Kawai T.; Kwon J. H.; Kim D.; Aida T. Construction of Segregated Arrays of Multiple Donor and Acceptor Units Using a Dendritic Scaffold: Remarkable Dendrimer Effects on Photoinduced Charge Separation. J. Am. Chem. Soc. 2006, 128, 10527–10532. 10.1021/ja063081t. 16895420

[ref13] Harvey P. D.; Stern C.; Gros C. P.; Guilard R. The Photophysics and Photochemistry of Cofacial Free Base and Metallated Bisporphyrins Held Together by Covalent Architectures. Coord. Chem. Rev. 2007, 251, 401–428. 10.1016/j.ccr.2006.06.009.

[ref14] Nakamura Y.; Aratani N.; Osuka A. Cyclic Porphyrin Arrays as Artificial Photosynthetic Antenna: Synthesis and Excitation Energy Transfer. Chem. Soc. Rev. 2007, 36, 831–845. 10.1039/b618854k. 17534471

[ref15] Satake A.; Kobuke Y. Artificial Photosynthetic Systems: Assemblies of Slipped Cofacial Porphyrins and Phthalocyanines Showing Strong Electronic Coupling. Org. Biomol. Chem. 2007, 5, 1679–1691. 10.1039/b703405a. 17520134

[ref16] Gust D.; Moore T. A.; Moore A. L. Solar Fuels via Artificial Photosynthesis. Acc. Chem. Res. 2009, 42, 1890–1898. 10.1021/ar900209b. 19902921

[ref17] Wasielewski M. R. Self-Assembly Strategies for Integrating Light Harvesting and Charge Separation in Artificial Photosynthetic Systems. Acc. Chem. Res. 2009, 42, 1910–1921. 10.1021/ar9001735. 19803479

[ref18] Lindsey J. S.; Bocian D. F. Molecules for Charge-Based Information Storage. Acc. Chem. Res. 2011, 44, 638–650. 10.1021/ar200107x. 21627067

[ref19] Pellegrin Y.; Odobel F. Molecular Devices Featuring Sequential Photoinduced Charge Separations for the Storage of Multiple Redox Equivalents. Coord. Chem. Rev. 2011, 255, 2578–2593. 10.1016/j.ccr.2010.12.017.

[ref20] Beletskaya I.; Tyurin V. S.; Tsivadze A. Y.; Guilard R.; Stern C. Supramolecular Chemistry of Metalloporphyrins. Chem. Rev. 2009, 109, 1659–1713. 10.1021/cr800247a. 19301872

[ref21] Kim D. Multiporphyrin Arrays: Fundamentals and Applications; Edited by Jenny Stanford Publishing, 2012; pp 1–777.

[ref22] Handbook of Porphyrin Science with Applications to Chemistry, Physics, Materials Science, Engineering, Biology and Medicine; Kadish K. M., Guilard R., Smith K. M., Eds.; World Scientific, 2010; Vol. 10, pp 1–533.

[ref23] Kocher L.; Durot S.; Heitz V. Control of the Cavity Size of Flexible Covalent Cages by Silver Coordination to the Peripheral Binding Sites. Chem. Commun. 2015, 51, 13181–13184. 10.1039/c5cc04972e. 26193927

[ref24] Schoepff L.; Kocher L.; Durot S.; Heitz V. Chemically Induced Breathing of Flexible Porphyrinic Covalent Cages. J. Org. Chem. 2017, 82, 5845–5851. 10.1021/acs.joc.7b00698. 28481531

[ref25] Djemili R.; Kocher L.; Durot S.; Peuronen A.; Rissanen K.; Heitz V. Positive Allosteric Control of Guests Encapsulation by Metal Binding to Covalent Porphyrin Cages. Chem.—Eur. J. 2019, 25, 1481–1487. 10.1002/chem.201980661. 30536482

[ref26] Zanetti-Polzi L.; Amadei A.; Djemili R.; Durot S.; Schoepff L.; Heitz V.; Ventura B.; Daidone I. Interpretation of Experimental Soret Bands of Porphyrins in Flexible Covalent Cages and in Their Related Ag(I) Fixed Complexes. J. Phys. Chem. C 2019, 123, 13094–13103. 10.1021/acs.jpcc.9b00742.

[ref27] Zanetti-Polzi L.; Djemili R.; Durot S.; Heitz V.; Daidone I.; Ventura B. Allosteric Control of Naphthalene Diimide Encapsulation and Electron Transfer in Porphyrin Containers: Photophysical Studies and Molecular Dynamics Simulation. Chem.—Eur. J. 2020, 26, 17514–17524. 10.1002/chem.202003151. 32845572

[ref28] Durot S.; Flamigni L.; Taesch J.; Dang T. T.; Heitz V.; Ventura B. Synthesis and Solution Studies of Silver(I)-Assembled Porphyrin Coordination Cages. Chem.—Eur. J. 2014, 20, 9979–9990. 10.1002/chem.201402047. 25042755

[ref29] Sánchez-Resa D.; Schoepff L.; Djemili R.; Durot S.; Heitz V.; Ventura B. Photophysical Properties of Porphyrinic Covalent Cages Endowed with Different Flexible Linkers. J. Porphyrins Phthalocyanines 2019, 23, 841–849. 10.1142/s1088424619500925.

[ref30] Nakamura T.; Ube H.; Shionoya M. Silver-Mediated Formation of a Cofacial Porphyrin Dimer with the Ability to Intercalate Aromatic Molecules. Angew. Chem., Int. Ed. 2013, 52, 12096–12100. 10.1002/anie.201306510. 24115370

[ref31] Kishi N.; Akita M.; Kamiya M.; Hayashi S.; Hsu H.-F.; Yoshizawa M. Facile Catch and Release of Fullerenes Using a Photoresponsive Molecular Tube. J. Am. Chem. Soc. 2013, 135, 12976–12979. 10.1021/ja406893y. 23957216

[ref32] ReactLab Equilibria 1.1; Jplus Consulting Pty Ltd., 2009.

[ref33] Malde A. K.; Zuo L.; Breeze M.; Stroet M.; Poger D.; Nair P. C.; Oostenbrink C.; Mark A. E. An Automated Force Field Topology Builder (ATB) and Repository: Version 1.0. J. Chem. Theory Comput. 2011, 7, 4026–4037. 10.1021/ct200196m. 26598349

[ref34] Bussi G.; Donadio D.; Parrinello M. Canonical Sampling through Velocity Rescaling. J. Chem. Phys. 2007, 126, 014101 10.1063/1.2408420. 17212484

[ref35] Hess B.; Bekker H.; Berendsen H. J. C.; Fraaije J. G. E. M. LINCS: A Linear Constraint Solver for Molecular Simulations. J. Comput. Chem. 1997, 18, 1463–1472. 10.1002/(sici)1096-987x(199709)18:12<1463::aid-jcc4>3.0.co;2-h.

[ref36] Darden T.; York D.; Pedersen L. Particle Mesh Ewald: An N·log(N) Method for Ewald Sums in Large Systems. J. Chem. Phys. 1993, 98, 10089–10092. 10.1063/1.464397.

[ref37] Amadei A.; Daidone I.; Aschi M. A General Theoretical Model for Electron Transfer Reactions in Complex Systems. Phys. Chem. Chem. Phys. 2012, 14, 1360–1370. 10.1039/c1cp22309g. 22158942

[ref38] Zanetti-Polzi L.; Aschi M.; Daidone I.; Amadei A. Theoretical Modeling of the Absorption Spectrum of Aqueous Riboflavin. Chem. Phys. Lett. 2017, 669, 119–124. 10.1016/j.cplett.2016.12.022.

[ref39] Aschi M.; D’Abramo M.; Ramondo F.; Daidone I.; D’Alessandro M.; Di Nola A.; Amadei A. Theoretical Modeling of Chemical Reactions in Complex Environments: The Intramolecular Proton Transfer in Aqueous Malonaldehyde. J. Phys. Org. Chem. 2006, 19, 518–530. 10.1002/poc.1051.

[ref40] Kadish K. M.; Lin X. Q.; Ding J. Q.; Wu Y. T.; Araullo C. A Reinvestigation of Silver Porphyrin Electrochemistry—Reactions of Ag(III), Ag(II) and Ag(I). Inorg. Chem. 1986, 25, 3236–3242. 10.1021/ic00238a029.

[ref41] Dang T. T.; Durot S.; Monnereau L.; Heitz V.; Barbieri A.; Ventura B. Highlight on the Solution Processes Occurring on Silver(i)-Assembling Porphyrins in the Presence of an Excess of Silver Salt. Dalton Trans. 2017, 46, 9375–9381. 10.1039/c7dt00974g. 28686279

[ref42] Horváth O.; Valicsek Z.; Harrach G.; Lendvay G.; Fodor M. A. Spectroscopic and Photochemical Properties of Water-Soluble Metalloporphyrins of Distorted Structure. Coord. Chem. Rev. 2012, 256, 1531–1545. 10.1016/j.ccr.2012.02.011.

[ref43] Harrach G.; Valicsek Z.; Horváth O. Water-Soluble Silver(II) and Gold(III) Porphyrins: The Effect of Structural Distortion on the Photophysical and Photochemical Behavior. Inorg. Chem. Commun. 2011, 14, 1756–1761. 10.1016/j.inoche.2011.08.003.

[ref44] Dorough G. D.; Miller J. R.; Huennekens F. M. Spectra of the Metallo-Derivatives of α,β,γ,δ-Tetraphenylporphine. J. Am. Chem. Soc. 1951, 73, 4315–4320. 10.1021/ja01153a085.

[ref45] Ballester P.; Claudel M.; Durot S.; Kocher L.; Schoepff L.; Heitz V. A Porphyrin Coordination Cage Assembled from Four Silver(I) Triazolyl-Pyridine Complexes. Chem.—Eur. J. 2015, 21, 15339–15348. 10.1002/chem.201502152. 26338089

