# Supplementary material for: Photophysical and Computational Insights into Ag(I) Complexation of Porphyrinic Covalent Cages Equipped with Triazoles-Incorporating Linkers
Source: J Phys Chem B. 2022 Apr 28;126(18):3450–9. doi: 10.1021/acs.jpcb.2c01111 (PMC9109141; doi:10.1021/acs.jpcb.2c01111)
Supplement: Supplementary file 1 — jp2c01111_si_001.pdf [file jp2c01111_si_001.pdf]

Supporting Information for

Photophysical and Computational Insights into Ag(I) Complexation of Porphyrinic Covalent Cages Equipped with Triazoles Incorporating Linkers

Daniel Sánchez-Resa,[a] *Isabella Daidone,*[b] *Ryan Djemili,*[c] *Sonia Adrouche,*[c] *Stéphanie Durot,*[c] *Valérie Heitz,*[*,c] *Laura Zanetti-Polzi,*[*,d] *Barbara Ventura*[*,a]

[a] Isituto ISOF-CNR, Via P. Gobetti 101, 40129 Bologna, Italy. email: barbara.ventura@isof.cnr.it

[b] Department of Physical and Chemical Sciences, University of L'Aquila, Via Vetoio (Coppito 1), 67010 L'Aquila, Italy.

[c] Laboratoire de Synthèse des Assemblages Moléculaires Multifonctionnels, Institut de Chimie de Strasbourg, CNRS/UMR 7177, Université de Strasbourg, France. email: v.heitz@unistra.fr

[d] CNR Institute of Nanoscience, Via Campi 213/A, 41125 Modena, Italy. email: laura.zanettipolzi@nano.cnr.it

Table of contents:

Additional spectroscopic data Pag. S2
Figure S1 Pag. S2
Figure S2 Pag. S2
Figure S3 Pag. S3
Chart S1 Pag. S3

Figure S4 Pag. S4
Figure S5 Pag. S5
Figure S6 Pag. S5
Figure S7 Pag. S6
Figure S8 Pag. S6
Additional computational data Pag. S7
Figure S9 Pag. S7
Figure S10 Pag. S7

Additional spectroscopic data:

- **2H-S-2H**

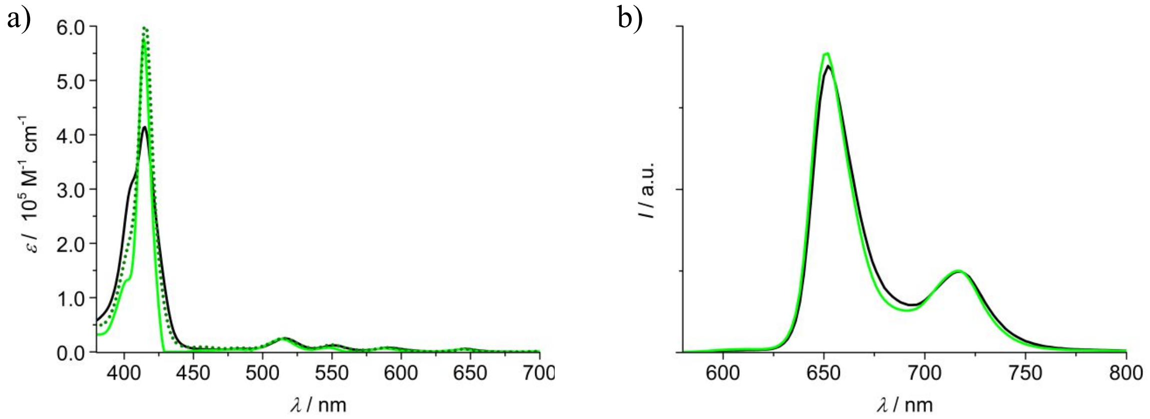

Figure S1. a) Experimental absorption spectrum of **2H-S-2H** (black, imposed in the fitting) and fitted absorption spectrum of the complex [(**2H-S-2H**)Ag_4]$^{4+}$ (green) in DCM/MeOH (9:1). The experimental absorption spectrum registered at the end of the titration is also reported for comparison purposes (olive dotted). b) Fitted emission spectra of **2H-S-2H** (black) and [(**2H-S-2H**)Ag_4]$^{4+}$ (green). The fittings were performed with the software Reactlab Equilibria by using a 1:4 (cage:Ag^+) binding model and Ag^+ as a non-absorbing and non-emissive species.

- **2H-L-2H**

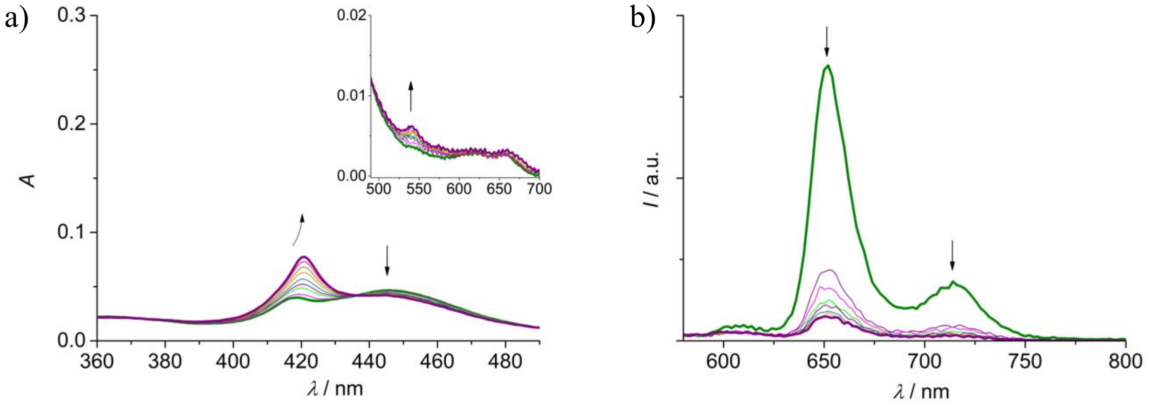

Figure S2. Absorption (a) and uncorrected emission (b) spectra (λ_{exc} = 434 nm, isosbestic point) of DCM/MeOH (9:1) solutions containing **2H-L-2H** (5.6 × 10^{-7} M) and increasing amounts of Ag(OTf) (30-400 equivalents). Inset of (a): amplification of the Q-bands region (490-700 nm). In (b) the y-axis scale is amplified by a factor 3 with respect to the scale of Figure 3b.

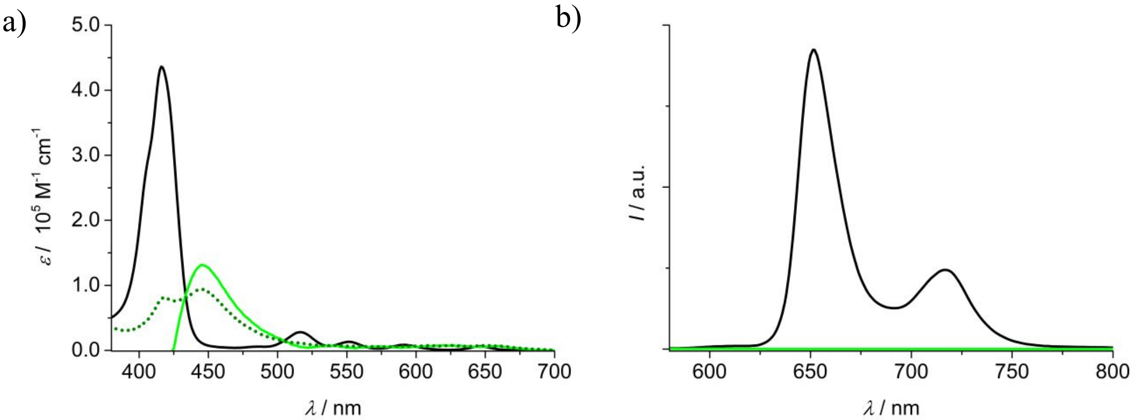

Figure S3. a) Experimental absorption spectrum of **2H-L-2H** (black, imposed in the fitting) and fitted absorption spectrum of the complex [(**2H-L-2H**)Ag_2]$^{4+}$ (green) in DCM/MeOH (9:1). The experimental absorption spectrum registered at the end of the first step (30 equiv.) of the titration is also reported for comparison purposes (olive dotted). b) Fitted emission spectra of **2H-L-2H** (black) and [(**2H-L-2H**)Ag_2]$^{4+}$ (green). The fitting was performed with the software Reactlab Equilibria by using a 1:2 (cage:Ag^+) binding model and Ag^+ as a non-absorbing and non-emissive species.

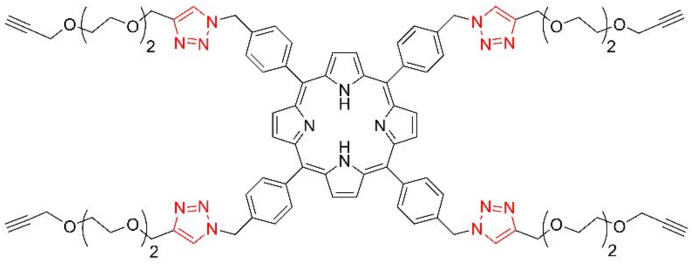

Chart S1. Structure of model **2H-alkyne**.

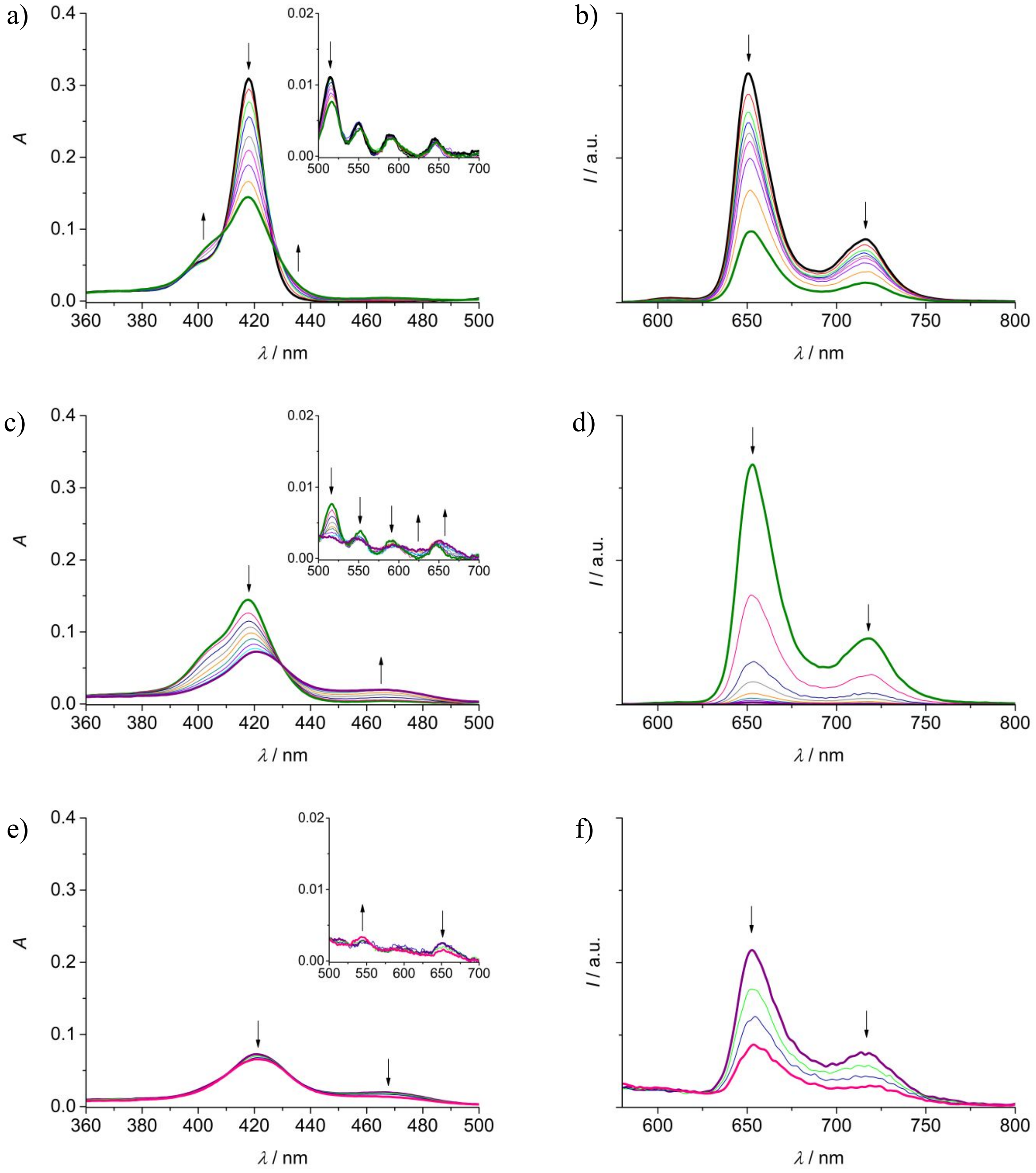

Figure S4. Absorption and uncorrected emission spectra of DCM/MeOH (9:1) solutions containing **2H-alkyne** (1.2×10^{-6} M) and increasing amounts of Ag(OTf): (a-b) 0-9 equivalents, λ_{exc} = 428 nm, isosbestic point; (c-d) 9-30 equivalents, λ_{exc} = 430 nm, isosbestic point; (e-f) 30-250 equivalents, λ_{exc} = 430 nm, isoabsorbing point. In (f) the y-axis scale is amplified by a factor 15 with respect to the scale of (d). Insets of (a,c,e): amplification of the Q-bands region (500-700 nm).

- **Zn-S-2H**

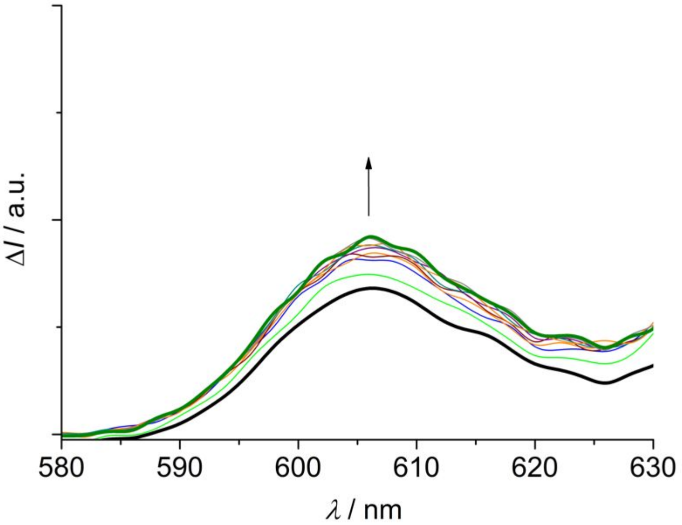

Figure S5. Emission spectra (λ_{exc} = 415.5 nm) of DCM/MeOH (9:1) solutions containing **Zn-S-2H** (5.5×10^{-7} M) and increasing amounts of AgOTf (0-40 equivalents). The spectra are expressed as differential intensity, calculated by subtracting, from the spectrum of the cage at each point of the titration, an emission spectrum of **2H-S-2H** normalized at 712 nm.

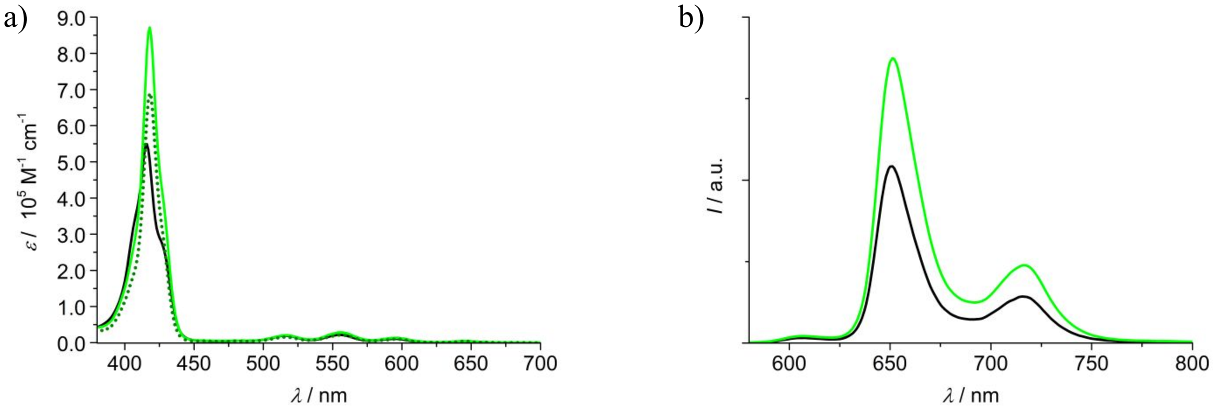

Figure S6. a) Experimental absorption spectrum of **Zn-S-2H** (black, imposed in the fitting) and fitted absorption spectrum of the complex [(**Zn-S-2H**)Ag_4]$^{4+}$ (green) in DCM/MeOH (9:1). The experimental absorption spectrum registered at the end of the titration is also reported for comparison purposes (olive dotted). b) Fitted emission spectra of **Zn-S-2H** (black) and [(**Zn-S-2H**)Ag_4]$^{4+}$ (green). The fittings were performed with the software Reactlab Equilibria by using a 1:4 (cage:Ag^+) binding model and Ag^+ as a non-absorbing and non-emissive species.

- **Zn-L-2H**

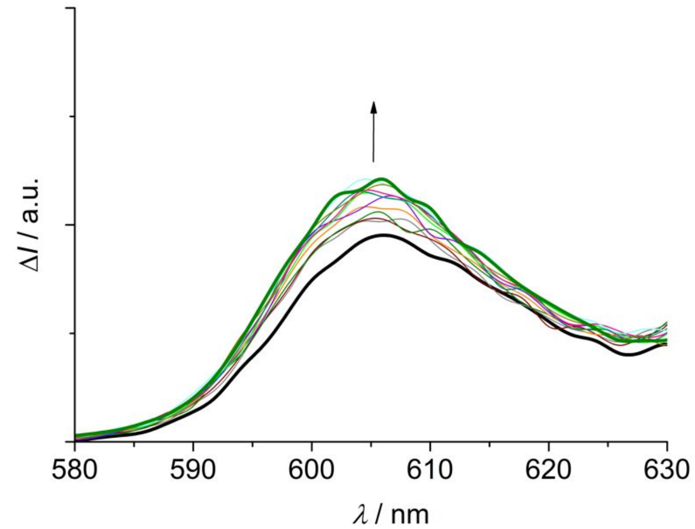

Figure S7. Emission spectra (λ_{exc} = 421 nm) of DCM/MeOH (9:1) solutions containing **Zn-L-2H** (6.5×10^{-7} M) and increasing amounts of AgOTf (0-40 equivalents). The spectra are expressed as differential intensity, calculated by subtracting, from the spectrum of the cage at each point of the titration, an emission spectrum of **2H-L-2H** normalized at 712 nm.

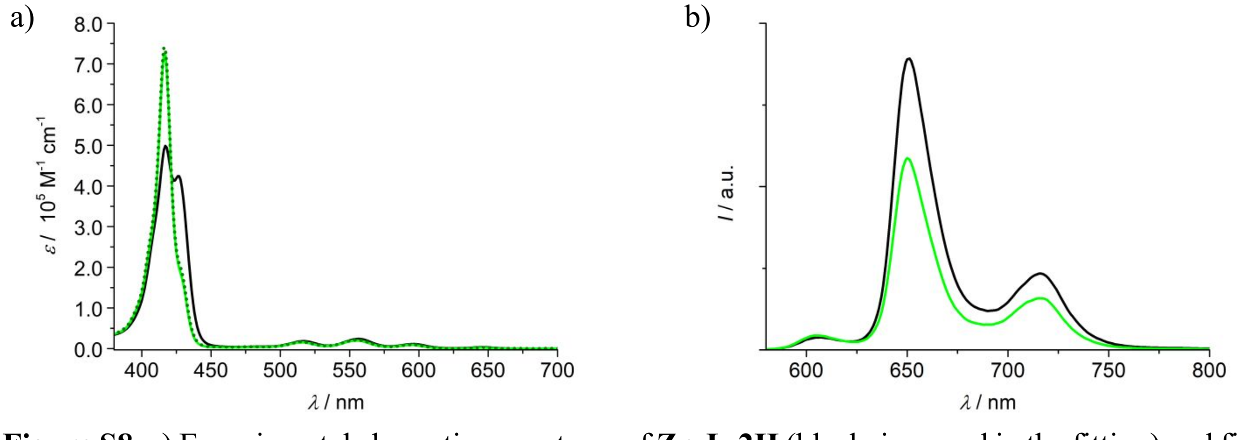

Figure S8. a) Experimental absorption spectrum of **Zn-L-2H** (black, imposed in the fitting) and fitted absorption spectrum of the complex $[(\textbf{Zn-L-2H})Ag_4]^{4+}$ (green) in DCM/MeOH (9:1). The experimental absorption spectrum registered at the end of the titration is also reported for comparison purposes (olive dotted). b) Fitted emission spectra of **Zn-L-2H** (black) and $[(\textbf{Zn-L-2H})Ag_4]^{4+}$ (green). The fittings were performed with the software Reactlab Equilibria by using a 1:4 (cage:Ag^+) binding model and Ag^+ as a non-absorbing and non-emissive species.

Additional computational data:

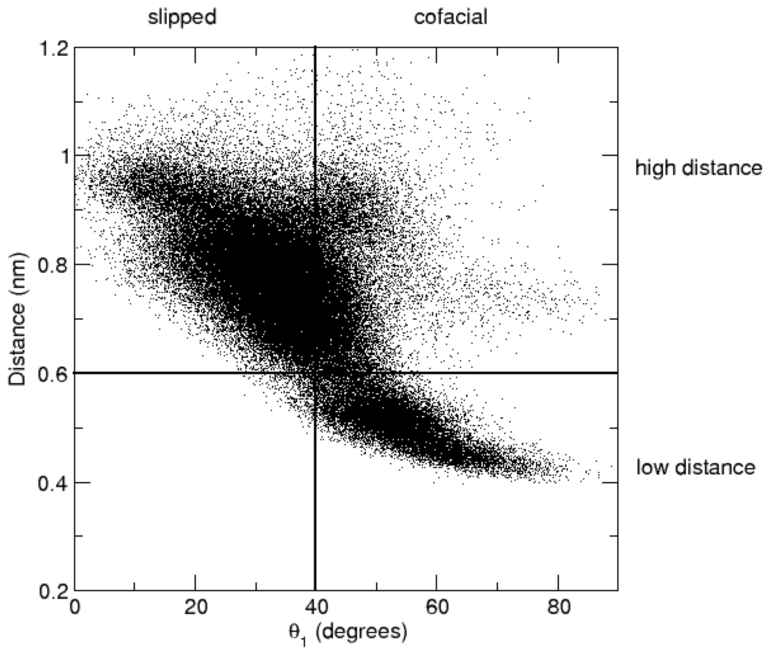

Figure S9. Bidimensional distribution of the distance between the centers of mass of the two porphyrin rings and θ1 in the MD simulation of **2H-S-2H**.

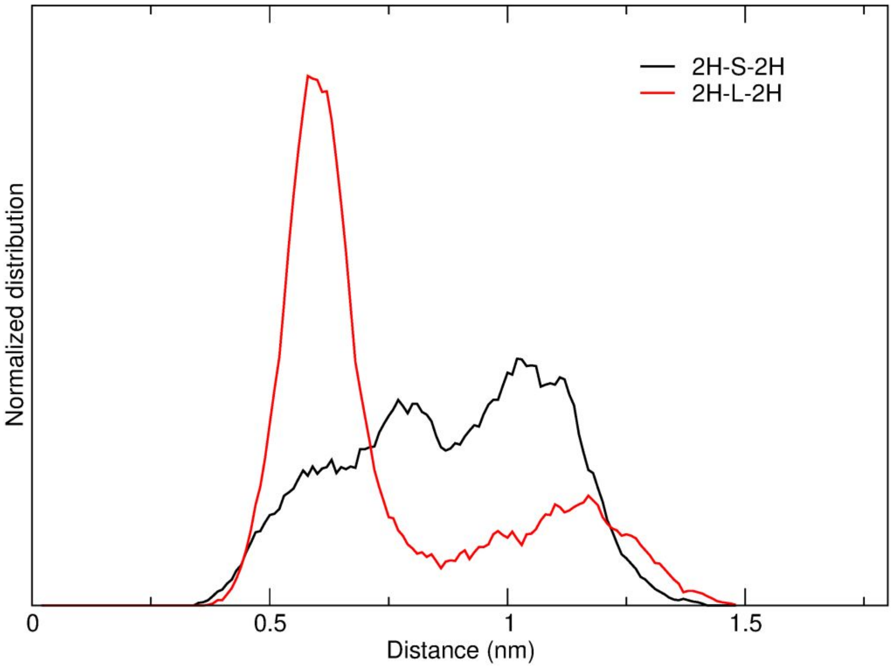

Figure S10. Normalized distribution of the distance between the two carbons connecting the phenyl and triazole rings of each linker in the MD simulations of **2H-S-2H** (black) and **2H-L-2H** (red). The curves are obtained by averaging the distributions of the distance obtained for each of the four linkers of the two cages.
